# Misdiagnosis and prevalence of rheumatological diseases in early inflammatory arthritis clinic: results from a UK single-centre retrospective cohort study

**DOI:** 10.1016/j.ero.2025.06.009

**Published:** 2025-07-21

**Authors:** Zina Mobarak, Muhammad Saad Aleem, Jamie Tulloch, Francesca Oram, Catinca Ciuculete, Fatima Saqib, Khaled Hasanein, Anne Barton, Pauline Ho, Ryan Malcolm Hum

**Affiliations:** 1 The Kellgren Centre for Rheumatology, Manchester Royal Infirmary, Manchester University NHS Foundation Trust, Manchester, UK; 2 Centre for Musculoskeletal Research, Division of Musculoskeletal and Dermatological Sciences, School of Biological Sciences, Faculty of Biology, Medicine and Health, The University of Manchester, Manchester, UK; 3 National Institute for Health and Care Research (NIHR) Manchester Biomedical Research Centre (BRC), The University of Manchester, Manchester, UK

## Abstract

**Objectives:**

Novel diagnostic tools have been developed to aid diagnosis and reduce misdiagnosis. However, the accuracy of these tools depends on estimates of disease prevalence. This study aimed to determine the prevalence of rheumatological diseases in a real-world cohort of patients and assess the rate of misdiagnosis at first presentation and follow-up.

**Methods:**

Patients with symptoms of inflammatory arthritis were referred by primary care. Rheumatologists made initial diagnoses, initiated treatment and revised diagnoses during follow-up. Clinical data including diagnoses were extracted from electronic patient records.

**Results:**

Over 2 years, 260 patients were seen. Most patients presented with peripheral joint symptoms (95%), and 23% had axial involvement. Psoriasis was present in 15%, inflammatory bowel disease in 4% and uveitis in 4%. Autoantibodies were positive for cyclic citrullinated peptide in 11% and rheumatoid factor in 12%.

Seventeen per cent (n = 45) of patients were misdiagnosed, with 8% (n = 20) initially diagnosed with 1 of the 5 most common rheumatological conditions but later found to have a noninflammatory disorder. Conversely, 7% (n = 17) initially received a diagnosis of an ‘other rheumatological disease’ but later had their diagnoses revised to 1 of the 5 most common rheumatological conditions.

**Conclusions:**

Disease prevalence is context-specific, and prevalence estimates in the intended setting should be determined prior to implementation of novel diagnostic tools. In real-world cohorts, noninflammatory diseases like chronic pain syndromes were more prevalent than in observational studies and registries. Misdiagnosis occurs in about one-fifth of patients, highlighting the benefits of novel diagnostic tools for earlier, more accurate diagnosis.

**WHAT IS ALREADY KNOWN ON THIS TOPIC**

• Novel diagnostic tools have and continue to be developed to help clinicians make more accurate diagnoses and reduce misdiagnosis; however, we know that several of these tools depend on accurate estimates of disease prevalence. Furthermore, understanding the burden of misdiagnosis is key to implementing these tools and assessing their benefit in the real world.

**WHAT THIS STUDY ADDS**

• This study determined that misdiagnosis occurred in 17% of patients over a 2-year period in a rheumatology clinic seeing patients with symptoms of inflammatory arthritis. Prevalence of diseases was found to be similar to other published real-world cohorts, but different to biobank and registry studies.

**HOW THIS STUDY MIGHT AFFECT RESEARCH, PRACTICE, OR POLICY**

• This study highlights the benefits of novel diagnostic tools for earlier, more accurate diagnosis which will affect research that seeks to apply and validate these tools in prospective and real-world cohorts.

Alt-text: Unlabelled box

## INTRODUCTION

Rheumatological diseases are often multisystem; however, involvement of the musculoskeletal system is common, with patients typically presenting to rheumatology services with joint symptoms [1,2]. Joint pain and swelling are frequently the initial presenting complaint for inflammatory arthritides. With an annual incidence of 80 to 100 adults in 100,000, inflammatory arthritis is common, with rheumatoid arthritis being the most commonly diagnosed form [2,3].

Early aggressive treatment of inflammatory arthritides is associated with improved outcomes [2,4,5]. Early arthritis clinics were first established in the 1980s to aid earlier identification and initiation of treatment [6], and the European League against Rheumatism guidelines on early arthritis emphasise early referral to rheumatology [7]. In the UK, early inflammatory arthritis (EIA) clinics commonly accept referrals from primary care in order to see patients with suspected EIA and to initiate treatment promptly. The National Institute for Health and Care Excellence guidelines state that the target for patients to be seen in the EIA clinic is within 3 weeks and for treatment to be initiated within 6 weeks [8]. Similarly, the British Society for Rheumatology National Early Inflammatory Arthritis Audit states that the target is for patients to be seen within 3 weeks from referral and for patients diagnosed with an inflammatory arthritis to start treatment with a disease-modifying antirheumatic drug (DMARD) within 3 months [9].

Although EIA clinics enable patients to be seen and assessed promptly, a key problem facing early treatment of arthritis is that the classification of inflammatory arthritis is often difficult given the considerable clinical and pathological overlap between conditions especially early on in the disease course [1,10]. Despite the overlap of patients presenting with similar symptoms, clinicians still come to a diagnosis using history, clinical assessment and investigations. However, these different diagnoses have different initial treatment strategies. For example, the first-line recommended treatment for gout would be nonsteroidal anti-inflammatory drugs [11], whereas a diagnosis of rheumatoid arthritis would require disease-modifying antirheumatic drugs as first line [12]. Therefore, misdiagnosis of patients first presenting to rheumatology services can result in delays in effective treatment, as patients may be given the wrong first-line treatment. This can lead to a lower chance of achieving remission, irreversible joint damage, comorbidities and associated mortality [2,4].

Previous studies have estimated the prevalence of different rheumatological diseases in rheumatology outpatient clinics [13,14]. Two previous studies report rheumatological diagnoses from real-world cohorts. The first study by Vanhoof et al [13] reported the diagnoses of 1097 patients seen in a rheumatology outpatient clinic in Belgium. The second study by Oguntona et al [14] reported the diagnoses of 472 patients seen in a rheumatology outpatient clinic in Nigeria. In contrast, 2 studies have reported the prevalence of rheumatological diagnoses in observational cohorts and biobanks. One study by Hum et al [15] reported the prevalence of rheumatological diagnoses in 1047 patients from the Norfolk Arthritis Register, which is a prospective observational cohort of patients with EIA. Another study by Knevel et al [16] reported the prevalence of rheumatological diagnoses in 243 patients from the Boston-based Partners HealthCare Biobank. However, there have not been any previously published studies reporting the prevalence of different rheumatological diseases in a real-world cohort of EIA patients in the UK.

There are many novel diagnostic tools being developed to aid the diagnosis of inflammatory arthritis; however, as these tools intend to improve diagnosis, the scope of misdiagnosis needs to be understood before implementation to be able to assess cost-effectiveness and value of such tools in the real world.

‘Misdiagnosis’ is a key concept when considering the utility of novel diagnostic tools such as polygenic risk scores. Patients are given provisional diagnoses when first seen in the rheumatology clinic and given the appropriate corresponding treatment. At follow-up, the diagnosis may be revised and changed as more information (such as patient history, response to initial treatment, test results) becomes available and it becomes apparent that the initial provisional diagnosis was incorrect. It may be considered that such patients were ‘misdiagnosed’ at their first appointment and may have benefitted from novel diagnostic tools, which may have helped the rheumatologist make the correct diagnosis earlier. However, no previous studies have investigated the rates of misdiagnosis in the EIA clinic.

The aim of this study was to determine the prevalence of rheumatological diseases in a real-world cohort of patients newly referred to a UK tertiary EIA clinic over a 2-year period and to determine the proportion of patients who are ‘misdiagnosed’ on first presentation and have their diagnosis revised at follow-up.

## METHODS

### Clinical cohort

At a tertiary rheumatology centre in Manchester, UK, a weekly EIA clinic operates in which patients with new presentations of suspected inflammatory arthritis based on clinical symptoms and physical examination are referred by primary and secondary care for assessment.

All EIA clinic appointments between September 2022 and 2024 were reviewed retrospectively using electronic patient records. Clinical details were tabulated including age, gender, ethnicity, smoking status, joint involvement (peripheral/axial), extra-articular manifestations of disease activity, autoantibody status, inflammatory markers and treatment initiated.

### Initial and final diagnoses

After initial assessment on the first visit, patients were given a provisional diagnosis by a rheumatology consultant or a speciality registrar. The initial diagnoses were recorded. Patients were given a follow-up appointment in the EIA clinic before reaching a final diagnosis. In order to enable comparison with previously published studies, clinical diagnoses were grouped into 6 categories: rheumatoid arthritis (RA), psoriatic arthritis (PsA), axial spondylarthritis (AxSpA), systemic lupus erythematosus (SLE) and others (divided into ‘simple musculoskeletal disorders and chronic pain syndromes’, and ‘other rheumatological diseases’)

### Statistical analysis

Descriptive statistics were used to summarise the data using R v.4.1.0 and RStudio v.1.4.1106.

### Ethics

No specific ethical approval was required or obtained for this analysis.

## RESULTS

### Patient characteristics

During the 2-year period, 260 patients were seen in the EIA clinic. Demographic and clinical characteristics are shown in Table 1. Within the cohort, the majority of patients were female (n = 176, 68%), with a mean age of 48 (SD 16), of self-reported white ethnicity (n = 117, 45%) and had never smoked (n = 153, 59%). In terms of articular and extra-articular manifestations of disease, the majority of patients had peripheral joint involvement (n = 247, 95%), with 23% (n = 60) having axial involvement, 15% having skin involvement (n = 39), 4% having eye involvement (n = 9), and 4% having gastrointestinal involvement (n = 10). In terms of treatment, 15% of patients seen were initiated on a DMARD (n = 38), with 10% receiving some form of corticosteroid (n = 26). The majority of patients had raised inflammatory markers, where the mean C-reactive protein was 9.1 (reference range 0-5 mg/L), and the mean erythrocyte sedimentation rate was 18.1 (reference range 1-7 mm/h) on initial presentation to the EIA clinic. In terms of autoantibodies, 11% (n = 28) of patients were anticyclic citrullinated peptide (CCP)-positive, and 12% (n = 31) were rheumatoid factor (RF)-positive.

**Table 1:** Demographic and clinical characteristics of patients seen in the EIA clinic on initial presentation

Characteristic	n = 260
Age, mean (SD)	47.6 (16.3)
Female sex, n (%)	176 (67.7%)
Ethnicity, n (%)	
White	117 (45.0%)
Asian	57 (21.9%)
Unknown	46 (17.7%)
Black	27 (10.4%)
Chinese	7 (2.7%)
Mixed	6 (2.3%)
Smoking, n (%)	
Current	37 (14.2%)
Ex	65 (25.0%)
Never	153 (58.8%)
Clinical involvement, n (%)	
Peripheral joint involvement	247 (95.0%)
Axial involvement	60 (23.1%)
Skin involvement	39 (15.0%)
Eye involvement	9 (3.5%)
Gastrointestinal involvement	10 (3.8%)
Treatment, n (%)	
DMARD	38 (14.6%)
IA injection	6 (2.3%)
IM steroid	14 (5.4%)
Oral steroids	6 (2.3%)
Inflammatory markers, mean (SD)	
CRP, mg/L	9.1 (20.6)
ESR, mm/h	18.1 (19.1)
Autoantibodies, n (%)	
CCP-positive	28 (10.8%)
RF-positive	31 (11.9%)

CCP, anticyclic citrullinated peptide; CRP, C-reactive protein; DMARD, disease-modifying antirheumatic drug; ESR, erythrocyte sedimentation rate; IA, intraarticular; IM, intramuscular; RF, rheumatoid factor.

### Diagnoses on first presentation and after follow-up in the EIA clinic

Provisional diagnoses for patients seen in the EIA clinic are shown in Table 2 [[13], [14]]. In the EIA clinic, 56% of patients (n = 146) seen were given an ‘other’ diagnosis after first presentation, with 126 (49%) being given a diagnosis of a simple musculoskeletal disorder and/or a chronic pain syndrome. Of the 5 rheumatological conditions commonly diagnosed in EIA clinics, the most common diagnosis on first presentation to EIA clinic was RA (15%, n = 40) followed by gout (11%, n = 29), PsA (8%, n = 20), AxSpA (5%, n = 12) and SLE (0.4%, n = 1).

**Table 2:** Clinical diagnoses in the EIA clinic cohort after first presentation and after final follow-up in the EIA clinic compared with other similar published cohorts

	EIA first (n = 260)	EIA final (n = 260)	NOAR G-PROB (n = 1047)	G-PROB partners biobank (n = 243)	Oguntona et al [14] (n = 472)	Vanhoof et al [13] (n = 1097)
RA	40 (15.4%)	37 (14.2%)	756 (72%)	114 (47%)	21 (4.4%)	110 (10%)
PsA	20 (7.7%)	10 (3.8%)	104 (10%)	22 (9%)	2 (0.4%)	22 (2%)
AxSpA	12 (4.6%)	9 (3.5%)	16 (2%)	7 (3%)	0 (0%)	33 (3%)
SLE	1 (0.0%)	0 (0%)	18 (2%)	7 (3%)	10 (0.3%)	4 (0.4%)
Gout	29 (11.1%)	25 (9.6%)	12 (1%)	22 (9%)	32 (6.8%)	22 (2%)
Other	158 (60.8%)	179 (68.8%)	141(14%)	71 (29%)	407 (88.1%)	906 (82.6%)
Simple Musculoskeletal Disorders and Chronic pain syndromes	130 (50.0%)	153 (58.8%)	75 (7%)		388 (95.3%)	735 (81.1%)
Other rheumatological diseases	28 (10.8%)	26 (10.0%)	66 (6%)		19 (4.7%)	171 (18.9%)

AxSpA, axial spondylarthritis; EIA, early inflammatory arthritis; G-PROB, genetic-probability tool; NOAR, Norfolk Arthritis Register; PsA, psoriatic arthritis; RA, rheumatoid arthritis; SLE, systemic lupus erythematosus.

After follow-up, the proportions of diagnoses varied from the proportions given on first presentation, but not the overall order and rank in terms of frequency. After follow-up, 69% had a diagnosis of an ‘other’ condition, with 160 (62%) having a diagnosis of a simple musculoskeletal disorder and/or chronic pain syndrome. Of the 5 rheumatological conditions commonly diagnosed in EIA clinics, the most common after follow-up remained RA (14%, n = 37), followed by gout (10% n = 25), PsA (4%, n = 10), AxSpA (3%, n = 8) and SLE (0.4%, n = 1).

### Patients ‘misdiagnosed’ on first presentation compared to final diagnosis at follow-up

Discrepancies between the diagnosis given on first presentation at the EIA clinic compared with the diagnosis given at follow-up were noted. It can be considered that these patients were ‘misdiagnosed’ on first presentation, as their diagnosis was revised/changed at follow-up after additional test results became available, time had elapsed for the disease to manifest and initial response to treatment had been noted. Within the EIA cohort, 45 patients (17%, n = 45/260) were ‘misdiagnosed’ and had their diagnoses changed at follow-up (Fig).

**Figure:**
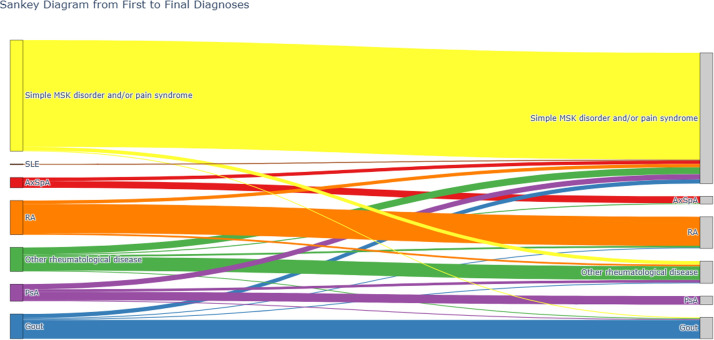
Sankey plot of initial and final diagnoses at follow-up, where patients ‘misdiagnosed’ on initial presentation will have their diagnosis changed at follow-up. AxSpA, axial spondylarthritis; MSK, musculoskeletal; PsA, psoriatic arthritis; RA, rheumatoid arthritis; SLE, systemic lupus erythematosus.

Of the 45 patients ‘misdiagnosed’, 28 had received a diagnosis of one of the 5 rheumatological conditions commonly diagnosed in EIA clinics. Ten patients initially diagnosed with PsA had their diagnoses changed, where the final diagnosis was a simple musculoskeletal disorder and/or pain syndrome in 6 instances, gout in 1 instance and another rheumatological disease in 3 instances. Seven patients initially diagnosed with gout had their diagnoses changed, where the final diagnosis was a simple musculoskeletal disorder and/or pain syndrome in 5 instances, RA in 1 instance and another rheumatological disease in 1 instance. Six patients initially diagnosed with RA had their diagnoses changed, where the final diagnosis was a simple musculoskeletal disorder and/or pain syndrome in 4 instances, and another rheumatological disease in 2 instances. Four patients initially diagnosed with AxSpA had their diagnoses changed, where the final diagnosis was a simple musculoskeletal disorder and/or pain syndrome in all 4 instances. One patient initially diagnosed with SLE had their diagnosis changed to a simple musculoskeletal disorder and/or pain syndrome. In total, 8% (n = 20) of the EIA clinic cohort were ‘misdiagnosed’ initially with one of the 5 rheumatological conditions and were subsequently given a diagnosis of a simple musculoskeletal disorder and/or pain syndrome at follow-up.

Of the 45 patients ‘misdiagnosed’, 17 had received a diagnosis of an ‘other’ disease on first presentation, which was then revised to 1 of the 5 rheumatological conditions commonly diagnosed in EIA clinics in 5 instances, including RA in 2 instances, gout in 2 instances and AxSpA in 1 instance. Of the remaining 12 instances, 4 initial diagnoses of simple musculoskeletal disorders and/or pain syndromes were changed to ‘other rheumatological diseases’ and 8 initial diagnoses of ‘other rheumatological diseases’ were changed to simple musculoskeletal disorders and/or pain syndromes.

### ‘Other rheumatological diseases’ diagnosed in the EIA clinic

Aside from the 5 most commonly diagnosed rheumatological conditions, the most common final diagnoses for the ‘other rheumatological diseases’ were undifferentiated connective tissue disease in 7 instances, reactive arthritis in 4 instances, primary Raynaud’s syndrome in 4 instances, sarcoidosis in 3 instances, enteropathic arthritis in 2 instances and erythema nodosum, dermatomyositis, polymyalgia rheumatica, Sjogren’s syndrome, systemic sclerosis and dupilumab-induced inflammatory arthritis in 1 instance each.

## DISCUSSION

In the first study to report the prevalence of rheumatological diseases and the rates of misdiagnosis in an EIA clinic in the UK, we found, first, that disease prevalence is highly context-specific. When comparing the prevalence of diseases in our cohort to previously published data, we found significant variation. In particular, our real-world cohort as well as previously published real-world cohort studies had a higher prevalence of noninflammatory conditions compared with observational cohort studies and biobanks [[13], [14], [15], [16]]. Second, we found that nearly a fifth of patients were initially ‘misdiagnosed’ and had their diagnosis revised at follow-up. This makes clear the scope of the issue, which may be addressed by novel diagnostic tools such as polygenic risk scores. However, given that we found prevalence was highly context-specific, our study highlights the critical importance of establishing accurate prevalence estimates prior to real-world application of tools such as G-PROB.

Our results align with previously published data on the characteristics of patients seen in the EIA clinic and reaffirm that the EIA clinic patient population is heterogeneous. Our results aligned with previously published studies in terms of the proportion of patients presenting with axial involvement (seen in 15% of patients in our cohort) and extra-articular involvement (skin involvement in 4%, inflammatory bowel disease in 4% and uveitis in 4%). In addition, we also provide data on the prevalence of CCP and RF antibodies as well as raised inflammatory markers. In this regard, our study highlights how a large proportion of patients referred to the EIA clinic are seronegative with slightly raised inflammatory markers, with only 11% of patients referred being CCP-positive. Additionally, we present data on treatments initiated in the EIA clinic, showing that one-tenth of patients receive corticosteroids in some form, while one-seventh are started on DMARDs.

In terms of disease prevalence in the EIA clinic population, we found that our results varied considerably from previously published cohorts. As our study examined a real-world cohort, our results unsurprisingly most closely align with 2 previously published studies from Belgium and Nigeria, which also reported disease prevalence in real-world rheumatology outpatient clinics [13,14]. However, disease prevalence in our study and the other previously published real-world cohorts varied considerably from those reported in Norfolk Arthritis Register, an observational cohort study, and from the Boston-based Partners HealthCare Biobank, which was used in the original study that developed the G-PROB tool [16]. The most obvious difference was in the prevalence of noninflammatory conditions, which had a higher prevalence in real-world cohorts. One possible explanation for this finding is selection bias in which patients with inflammatory conditions are more likely to join prospective observational studies and to consent to biobanking. Overall, our study highlights how disease prevalence is highly context-specific and that prevalence in real-world cohorts varies considerably compared with prospective studies and biobanks. Therefore, studies to estimate prevalence within the intended setting are necessary before applying tools like G-PROB whose performance relies on accurate prevalence estimates.

For the first time, our study reports that ‘misdiagnosis’ occurs in as many as one-fifth of patients seen in the EIA clinic. This has implications for future studies and research, which seek to develop and apply novel diagnostic tools, as it serves as a benchmark and highlights how much scope there is for improvement. In particular, 2% of patients who were initially diagnosed with an ‘other rheumatological diseases’ had their diagnoses revised to one of the 5 most common rheumatological conditions. For these patients, had the correct diagnosis been determined earlier, the appropriate treatment would likely have been initiated sooner, potentially improving long-term outcomes for these patients. Conversely, 8% of patients were initially ‘misdiagnosed’ with 1 of the 5 most common rheumatological conditions and subsequently had their diagnoses revised to a simple musculoskeletal disorder and/or pain syndrome at follow-up. For these patients, had the correct diagnosis been determined earlier, it is possible that exposure to ineffective and potentially harmful treatments such as corticosteroids and DMARDs could have been reduced.

There are several limitations to our study. First, our study relied on clinical diagnoses recorded in clinical notes as opposed to classification criteria. Although this approach reflects real-world practice, there is variation in how different clinicians arrive at different diagnoses. As the patients in our study were seen in a single centre with multiple clinicians, the variation in clinical practice will have an effect on the prevalence of different diagnoses we report in this study. Justification for diagnoses was not recorded, and therefore, adjudication of diagnoses was not possible. Second, referral criteria from primary care and other factors affecting primary care will influence the types of patients who are referred and seen in the EIA clinic. For example, when there are pressures affecting access to primary care, such as increased waiting times or staff shortages, patients may have difficulty being seen and referred, which ultimately leads to delays in being seen in secondary care outpatient clinics. However, to address this limitation, our study evaluated patients seen and referred over 2 years to minimise the impact that any short-term disturbances will have on referrals from primary care. Third, variations in clinical practice in different countries will affect prevalence. For example, in the UK, gout is primarily managed by primary care and is not typically referred to secondary care; however, in other countries, this may not be the case.

The concept of misdiagnosis is also complex. A provisional diagnosis made initially may be appropriate at the time based on the information available. The fact that the diagnosis is revised or changed later may not necessarily indicate a mistake. Moreover, even if the diagnosis is changed, this does not always imply that the initial treatment plan would have been different. For instance, a change from a diagnosis of RA to PsA would not drastically alter the initial treatment approach as both conditions are initially treated with DMARDs. Conversely, a change in diagnosis from AxSpA to PsA could have altered the initial treatment approach, as AxSpA is managed with nonsteroidal inflammatory drugs initially as opposed to DMARDs. Most likely, the most important form of ‘misdiagnosis’ is when diagnoses are changed from noninflammatory conditions to inflammatory conditions or vice versa as these would have more significant treatment implications.

Although our study is a large cohort study over 2 years and is the first to describe and report on the concept of ‘misdiagnosis’ in the EIA clinic setting, it remains to be seen if addressing misdiagnosis through novel diagnostic tools such as G-PROB will improve outcomes for patients. Our study highlights how future studies that seek to apply novel diagnostic tools requiring accurate prevalence estimates, such as G-PROB, will need to determine prevalence estimates in their intended setting and population prior to application.

In conclusion, we describe a large real-world cohort of patients from an EIA clinic, in which disease prevalence was similar to other previously published real-world cohorts, but different to published cohorts from prospective observational studies and biobanks. Additionally, we found that misdiagnosis occurred in a fifth of patients, highlighting the scope for novel diagnostic tools such as G-PROB to improve diagnostic accuracy.

## Competing interests

RMH has received honorariums from Abbvie, Novartis and UCB.
